# The Present Raid on the Uterus

**Published:** 1868-01-01

**Authors:** 


THE PRESENT RAID ON THE UTERUS.—The following extract from the address of Dr. W. D. Buck, before the New Hampshire Medical Society, is copied from the St. Louis *Medical Reporter:* "The uterus is a harmless, inoffensive little organ, stowed away in a quiet little place. Simply a muscular organ, having no function to perform save at certain periods of life, but furnishing a capital field for surgical operations, and is now-a-days subject to all sorts of barbarity from surgeons anxious for notoriety. Had dame Nature forseen this, she would have made it iron-clad. What with burning and cauterizing, cutting and slashing, and splitting, and skewering, aud pessarying, the old fashioned womb will cease to exist, except in history. The Transactions of the National Medical Association for 1864, has figured in 123 different kinds of pessaries, embracing every variety, from a simple plug to a patent threshing machine, which can only be worn with the largest hoops. They look like the drawings of turbine water-wheels, or a leaf from a work on Entomology. Pessaries, I suppose, are sometimes useful, but there are more than there is necessity for. I do think this filling up the vagina with such traps, making a Chinese toy-shop of it, is outrageous. Hippocrates said he never would recommend a pessary to procure abortion, nay, he swore he never would. Were he alive now he would never recommend one at all. If there were fewer abortions there would be fewer pessaries, and if there were fewer pessaries there would be fewer abortions. Our grandmothers never knew they had wombs, only as they were reminded of it by a healthy fœtus; which, by the by, they always held on to. Now-a-days, even our young women must have their wombs shoved up, and if a baby accidentally gets in by the side of the machinery, and finds a lodgment in the uterus, it may, perchance, have a knitting-needle stuck in it eyes before it has any. It is the easiest thing in the world to introduce a speculum, and pretend to discover ulceration of the os, and subject a patient to this revolting manipulation once or twice a week, when there is, in fact, nothing the matter. By some practitioners, all diseases which occur in the female are attributed to the uterus. In this class are especially to be included all such as make of the abnormal conditions of the uterus a specialty."

---

Rush Medical College — Spring Course of Instruction. The spring course of instruction in this institution will commence on Monday, the 2nd day of March ensuing, and extend through three months. There will be daily recitations and lectures, together with unusual advantages for clinical instruction at the various hospitals of the city and the college dispensary. Details will be given in the next number of the Journal. Full information can be obtained by addressing the Secretary of the college. See advertisement. Prof. Blaney will open his school of instruction in analytical and applied chemistry at the same time, in the laboratory of the college.

